# Inter-observer agreement of baseline whole body MRI in multiple myeloma

**DOI:** 10.1186/s40644-020-00328-9

**Published:** 2020-07-14

**Authors:** James Croft, Angela Riddell, Dow-Mu Koh, Kate Downey, Matthew Blackledge, Marianne Usher, Kevin Boyd, Martin Kaiser, Christina Messiou

**Affiliations:** 1 The Institute of Cancer Research, London, UK; 2 The Royal Marsden Hospital, London, UK

**Keywords:** Multiple myeloma, Magnetic resonance imaging, MRI, Diffusion weighted imaging, Bone disease, Inter-observer agreement

## Abstract

**Background:**

Whole body magnetic resonance imaging (MRI) is now incorporated into international guidance for imaging patients with multiple myeloma. The aim of this study was to investigate inter-observer agreement of triple reported baseline whole-body MRI in myeloma and highlight potential pitfalls.

**Methods:**

Fifty-seven patients with symptomatic myeloma at first presentation or relapse and planned for autologous stem cell transplant were included. All patients completed baseline whole body MRI within 2 weeks prior to starting treatment. Each scan was reported independently by 3 radiologists using a defined scoring system. Differences in observer scores were compared using analysis of variance (ANOVA) and inter-observer agreement assessed using intra class correlation coefficient (ICC).

**Results:**

There was no significant difference in mean observer scores for whole skeleton and ICC demonstrated excellent inter-observer agreement at 0.91. ICC varied between skeletal regions with spine, pelvis and ribs showing good inter-observer agreement, whereas skull and long bones were moderate. Scans with variation in observer scores were re-examined and cause of discrepancies identified. This information was used to describe potential anatomical pitfalls in reporting .

**Conclusion:**

Whole-body MRI has excellent inter-observer agreement in reporting symptomatic myeloma at baseline. Inter-observer agreement varied between skeletal regions highlighting specific areas of difficulty.

## Background

Magnetic resonance imaging (MRI) has higher specificity and sensitivity in the detection of focal lesions in multiple myeloma when compared with x-ray, computed tomography (CT) and Fluorodeoxyglucose (FDG) positron emission tomography (PET)-CT [1–4]. It can also detect myeloma infiltration within the bone marrow before the development of cortical bone destruction [5]. This provides prognostic information, as more than one focal lesion is associated with higher risk of disease progression [6, 7]. If disease can be detected early, and patients stratified and treated according to clinical risk, survival advantages are conferred [7–12]. MRI is therefore the gold standard imaging technique for assessment of bone marrow involvement in myeloma. The presence of > 1 focal lesion of at least 5 mm is considered evidence of symptomatic disease requiring treatment as per the International Myeloma Working Group (IMWG). Whole body (WB) MRI is also recommended by the IMWG for all patients with suspected myeloma and negative/inconclusive CT and is offered as an option for bone marrow imaging by the European Society for Medical Oncology guidelines [6, 13, 14]. In the UK WB MRI is recommended as first line imaging for all patients with a suspected new diagnosis of myeloma [15].

WB MRI has shown particular value in myeloma due to excellent image contrast between normal and diseased bone marrow. This has translated into improved sensitivity of lesion detection when compared with conventional MRI techniques [5]. It also has the unique ability to quantify differences in bone marrow through measurement of apparent diffusion coefficient (ADC). This has been shown to differentiate normal from myeloma infiltrated bone marrow with a sensitivity of 90% and specificity of 93% but can also be used to quantify response to treatment [5, 16, 17]. Recently the Myeloma Response Assessment and Diagnosis System (MY-RADS) was published outlining recommendations for standardised acquisition and reporting [18].

Data regarding the visual inter-observer agreement of WB MRI in myeloma is limited to a small series. While shown to be superior to that of skeletal survey, specific anatomical areas such as the skull and ribs were shown to be more challenging [2, 17]. We therefore investigated inter-observer variation of triple reported WB MRI in a prospective study.

## Materials and methods

This was a single centre prospective study carried out in accordance with the Declaration of Helsinki (1996), with local Committee for Clinical Research and national Ethics Committee approval. Patients gave written consent to enter the study.

### Study population

Fifty-seven patients with symptomatic myeloma as per IMWG criteria [19] completed WB MRI including diffusion weighted (DW) MRI sequences, within 2 weeks prior to starting treatment between November 2015 and February 2018. Patients included had new presentation or first relapse of myeloma and were planned for autologous stem cell transplant at the Royal Marsden Hospital. Exclusion criteria were MRI incompatible metal implants, claustrophobia or the diagnosis of other malignancies within the past 5 years.

### Image acquisition

WB MRI studies were performed using an Avanto 1.5 T system (Siemens, Erlangen, Germany) as per the MY-RADS recommendations [18]. All subjects were scanned supine with arms by their sides. Coil elements were positioned from skull vertex to knees. Sagittal T1-weighted images (TR 590 ms, TE 11 ms, FOV 400 mm, slice thickness 4 mm), and T2-weighted images (TR 2690 ms, TE 93 ms, FOV 400 mm, slice thickness 4 mm) of the spine were acquired, followed by axial DW sequences (single-shot double spin echo echo-planar technique with STIR fat suppression in free breathing) using b-values of 50 and 900 s/mm^2^ applied in 3 orthogonal directions and combined to the isotropic trace images. DW images were acquired in multiple contiguous stations of 50 slices per station (slice thickness 5 mm, no gap, FOV 430 mm, phase direction AP, parallel imaging (GRAPPA) factor 2, TR 14800 ms, TE 66 ms, inversion time (TI) 180 ms, voxel size 2.9 mm × 2.9 mm × 5 mm, number of signal averages 4, matrix 150 × 150, bandwidth 1960 Hz per pixel). Axial T1-weighted Vibe Dixon 3D gradient echo breath-hold sequences (52 slices per slab, FOV 470 mm, TR/TE 7/2.38, 4.76 ms, flip angle 30, matrix 192 × 192) were also acquired, matching the acquisition stacks and partition thickness to the DW images. No intravenous gadolinium contrast was used.

### Image analyses

Images were scored independently by 3 radiologists (> 8 years of experience) based on a previously described WB DW score [2, 17]. Focal disease of each skeletal region (cervical spine, dorsal spine, lumber spine, pelvis, long bones, skull, ribs/other) was scored (3, 2, 1) for number (> 20, 10–20, < 10) and size (> 20,10–20, < 10 mm) of lesions respectively.

### Statistical analyses

One-way analysis of variance (ANOVA) was used to compare the mean difference in observer scores for whole skeleton and individual skeletal regions. Tukey Honest Significant Differences (Tukey HSD) was used to perform multiple pairwise comparisons of mean scores between each observer if ANOVA was consistent with a significant difference. A two-sided *P*-value of ≤0.05 was considered statistically significant. Inter-observer agreement was described using the intra class correlation coefficient (ICC). ICC estimates and corresponding 95% confident intervals were calculated using R package psych, based on two-way mixed effects, consistency, and single rater measurement. An ICC of < 0.5 was considered poor, 0.5–0.75 moderate, 0.75–0.9 good and > 0.9 excellent as previously reported [20].

## Results

A total of 57 patients were included in his study (32 male, 25 female, age range 31–71). Of these 45 were newly diagnosed and 12 at first relapse. All patients at first relapse achieved > 18 months progression free survival from previous transplant. Induction regimens prior to first transplant involved triplet combinations that included proteasome inhibitor (PI) and immunomodulatory (IMiD) in 75%, IMiD only (17%) and PI only (8%). 75% of patients proceeded successfully to planned autologous stem cell transplant. Patient demographics can be seen in Table 1.

**Table 1:** Patient demographics at study baseline

Sex, n (%)	Male	32 (56)
	Female	25 (44)
Mean age, years (range)		58 (31–71)
Isotype, n (%)	IgA	4 (7)
	IgG	33 (58)
	LCO	3 (23)
	NS	3 (5)
	Unknown	3 (5)
Time-point, n (%)	Presentation	45 (79)
	1st Relapse	12 (21)

*LCO* Light chain only, *NS* Non secretory, *PP* Paraprotein, *SFLC* Serum free light chain

### WB DW scores

**Fig. 1:**
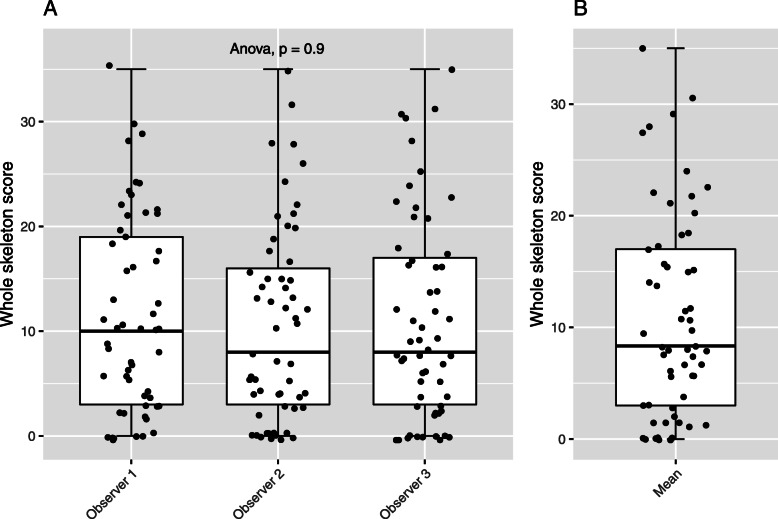
Whole skeleton scores (**a**) per observer, (**b**) mean scores

Distribution of bone disease was varied with whole skeleton scores ranging from 0 to 35. The mean score per skeletal region was lowest in the cervical spine (0.72) and highest in the pelvis (2.29). Distribution of mean whole skeleton scores per patient is shown in Fig. 1.

Mean observer scores for whole skeleton and individual skeletal regions are shown in Table 2 and comparison of whole skeleton scores per observer is demonstrated in Fig. 1. There was no significant difference between mean observer scores for whole skeleton or individual skeletal regions suggesting high inter-observer agreement. Pairwise comparison of observers also confirmed no significant difference in mean scores.

**Table 2:** Mean, standard deviation (SD) and range of combined scores for size and number of focal lesions per skeletal region. Statistical difference calculated using ANOVA

	Observer 1			Observer 2			Observer 3			
Region	Mean	SD	Range	Mean	SD	Range	Mean	SD	Range	ANOVA (*p* value)
Cervical Spine	0.77	1.34	0–5	0.68	1.27	0–4	0.65	1.34	0–6	0.88
Dorsal Spine	1.88	1.96	0–6	1.53	1.95	0–6	1.67	1.92	0–6	0.63
Lumbar Spine	1.39	1.68	0–6	1.32	1.69	0–5	1.33	1.76	0–6	0.95
Pelvis	2.35	2.22	0–6	2.21	2.41	0–6	2.30	2.25	0–6	0.93
Long Bones	1.95	1.87	0–6	1.91	2.12	0–6	2.05	2.02	0–6	0.97
Skull	0.67	1.30	0–4	0.98	1.38	0–4	0.72	1.25	0–4	0.55
Ribs	2.19	2.34	0–6	1.77	2.29	0–6	2.18	2.35	0–6	0.39
Whole Skeleton	11.19	9.43	0–35	10.40	9.35	0–35	10.89	9.61	0–35	0.90

The ICC [20] for whole skeleton and individual skeletal regions are shown in Table 3. There was excellent inter-observer reliability overall with whole skeleton ICC 0.91 (95% CI 0.87–0.94). Spine, pelvis and ribs all showed good inter-observer reliability with ICC ranging from 0.79–0.87, whereas long bones and skull were moderate. The ICC for the skull was 0.62 [95% CI 0.51–0.72] indicating worse inter-observer reliability compared to other skeletal regions, this is consistent with previous reports comparing MRI to skeletal survey [2].

**Table 3:** Intraclass correlation coefficient between observer scores per skeletal region

Region	ICC	95% CI
Cervical Spine	0.84	0.78–0.89
Dorsal Spine	0.85	0.80–0.90
Lumbar Spine	0.87	0.81–0.91
Pelvis	0.79	0.72–0.85
Long Bones	0.74	0.65–0.81
Skull	0.62	0.51–0.72
Ribs	0.82	0.76–0.88
Whole skeleton	0.91	0.87–0.94

*CI* Confidence intervals

## Discussion

This study investigated inter-observer agreement of WB MRI for baseline assessment of myeloma related bone disease in symptomatic patients at presentation or first relapse. Using ICC we demonstrate overall excellent inter-observer reliability on a simple scoring system, based on the number and size of focal lesions detected. When compared with previous studies, the ICC values were superior [2], which likely reflects growing expertise and knowledge of the technique. This is further highlighted by lack of significant difference in mean observer scores, an observation Giles et al. were previously unable to demonstrate [17]. With the exception of the skull, our ICC values were also consistently higher than those previously reported for skeletal survey [2], consolidating evidence for the superiority of WB MRI in the assessment of myeloma related bone disease.

**Fig. 2:**
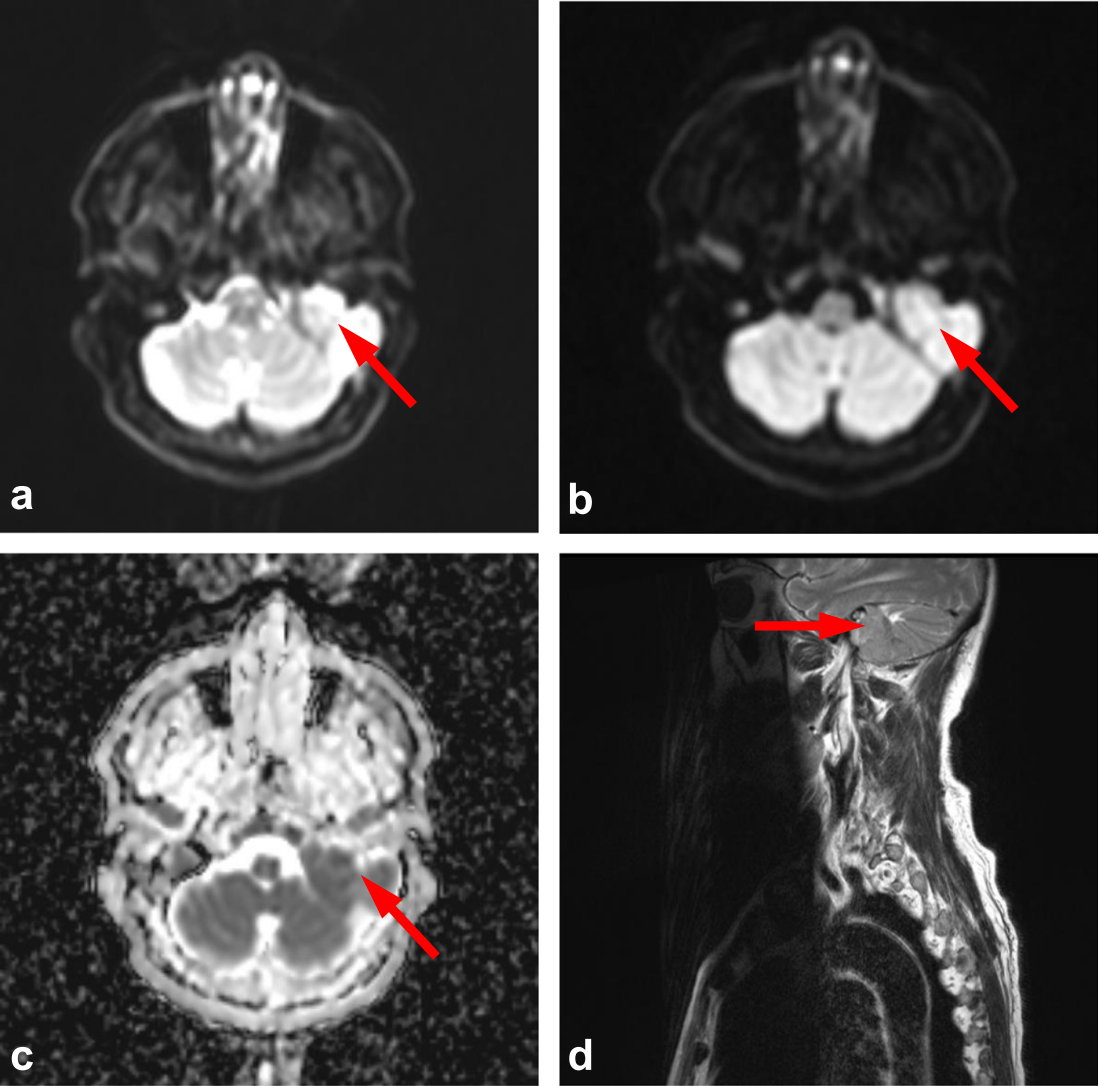
Base of skull lesion. Observer score variation within the skull due to large base of skull lesion (arrows) shown by (**a**) axial b50 DW MRI, **b** axial b900 DW MRI, **c** corresponding ADC map and (d) sagittal T2 weighted MRI of upper spine. Both brain and skull lesion have high diffusion signal (**a-b**) with low ADC (**c**), due to close proximity one observer reported images as normal

**Fig. 3:**
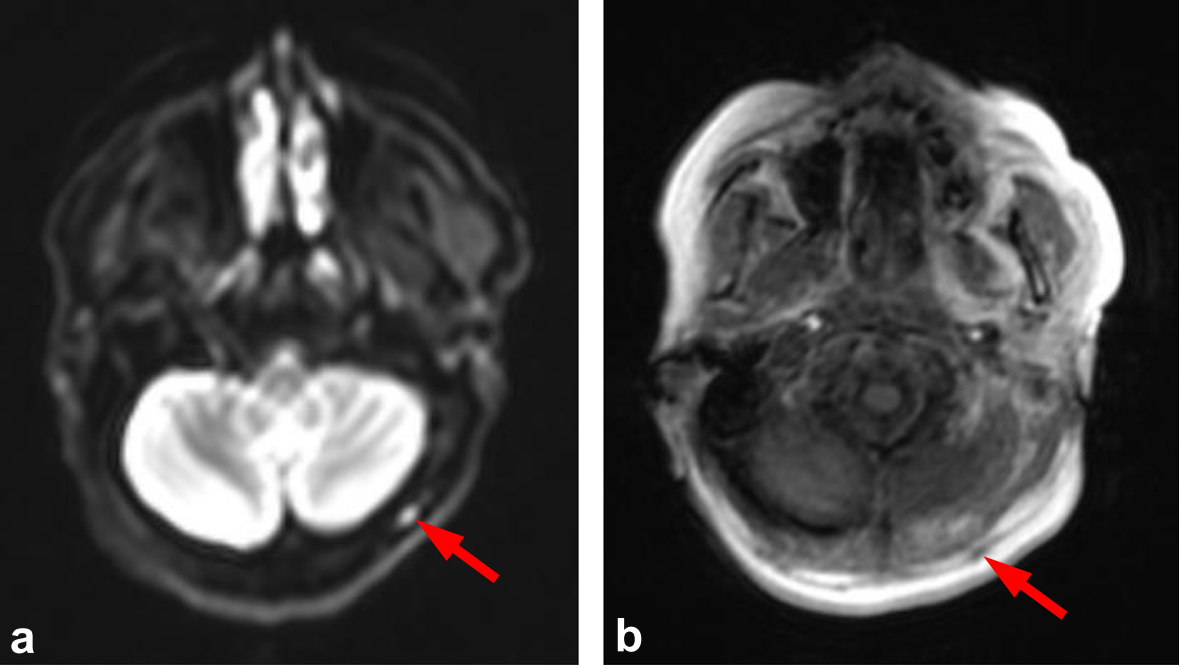
Thickening of subcutaneous facia. Observer score variation within the skull due to thickening of subcutaneous fascia (arrows) shown by (**a**) axial b900 DW MRI and (**b**) axial in-phase Dixon MRI. High diffusion signal leads to a false positive report of a skull lesion (arrow a), in-phase Dixon images show the lesion to not be within the skull and instead relate to thickening of subcutaneous facia (arrow b). Synonymous to this we also found sub-occipital lymph nodes can lead to false positive reporting of focal lesions within the skull

**Fig. 4:**
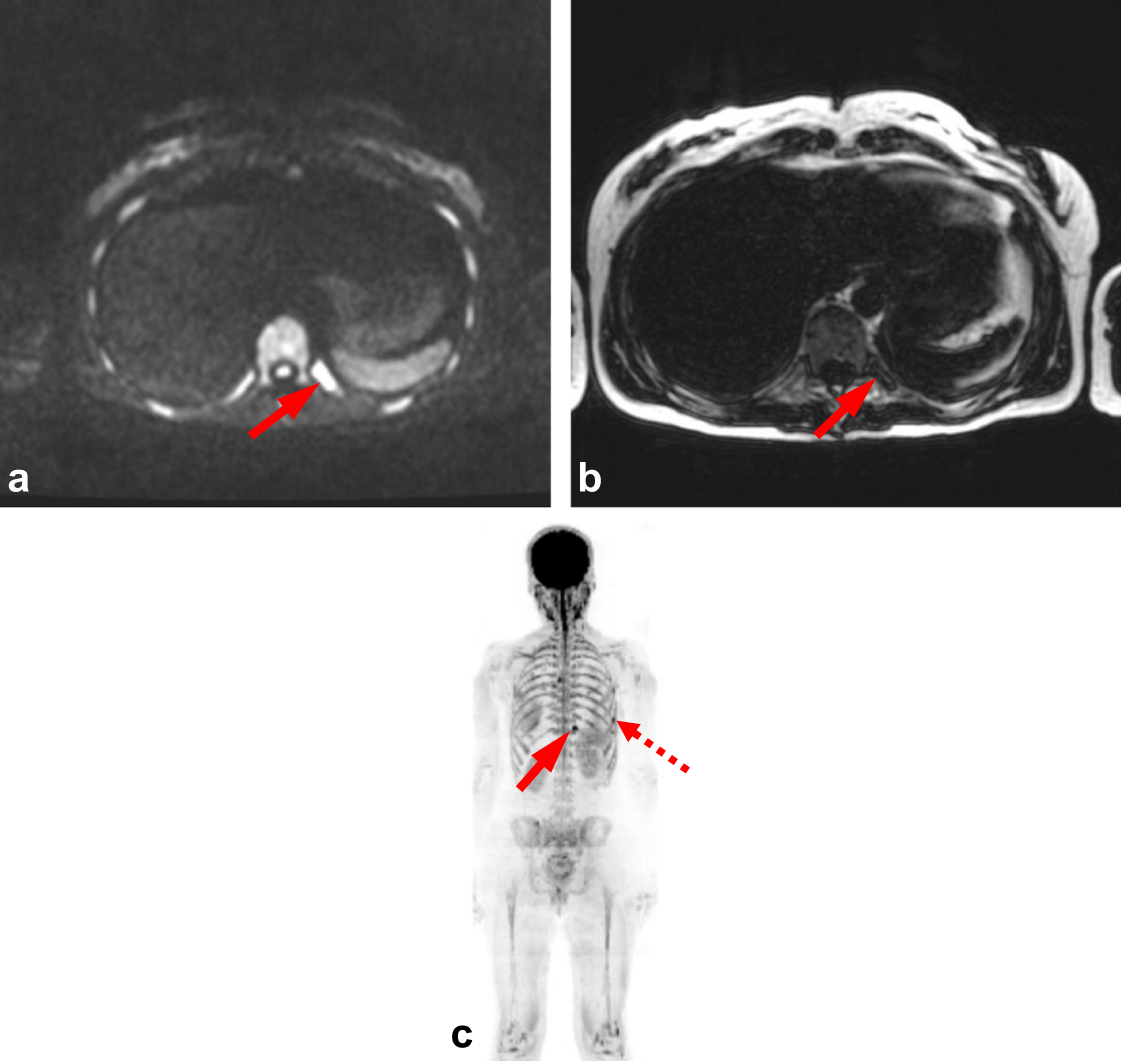
Focal lesion of ribs. Observer score variation within the ribs due to focal lesion (arrows) superimposed on diffuse marrow infiltration, shown by (**a**) axial b900 DW MRI, **b** axial fat-only Dixon and (**c**) b900 maximum intensity projection (MIP). Diffuse marrow infiltration results in high diffusion signal throughout ribs (**a**, **c**) which conceals superimposed focal lesion (arrow a). Fat only Dixon and MIP facilitate focal lesion differentiation (arrows b, c). Note MIP also demonstrates second focal lesion (dashed arrow c)

Variation between skeletal regions suggests that certain anatomical sites can be more challenging to score. Consistent with previous studies this was most notable in the skull, which is likely due to difficulties in interrogating relatively small marrow volume against adjacent high diffusion signal of the brain (Figs. 2 and 3) [5]. This limitation is paralleled in PET-CT where high FDG uptake of the brain also leads to difficulty in reporting adjacent bone lesions. Conversely, false positive results can occur with plain film of the skull due to venous lakes and arachnoid granulations [5]. Marrow assessment in the femora is also widely acknowledged to be challenging as areas of red marrow regeneration in the proximal femora can appear hypercellular mimicking disease and this uncertainty was reflected in a moderate ICC (0.74). Figure 4 demonstrates a focal rib lesion superimposed on diffuse marrow infiltration. Diffuse high signal throughout the ribs caused one observer to miss the focal lesion. Guidance from the IMWG advises anti-myeloma therapy for patients with> 1 focal lesion of > 5 mm. Therefore, false positive or negative reporting of any focal lesions could have significant clinical impact, highlighting the importance of examples we report. Knowledge and identification of such pitfalls are important to facilitate education and improve reporting accuracy.

Although the mixed cohort of patients with a new diagnosis of myeloma and relapsed myeloma reflects real world application, the imbalance of the classes (45 newly diagnosed and 12 relapsed) negates separate analysis. Background changes in bone marrow post treatment could make assessment more challenging and this has not been explored.

## Conclusion

WB MRI has excellent overall inter-observer reliability for the visual assessment of bone disease in symptomatic patients with multiple myeloma at presentation or first relapse. As with all imaging modalities, pitfalls in visual reporting exist and by reporting our own experience we hope to facilitate ongoing improvement to enable effective utilisation of the technique.

## Data Availability

The datasets used and analysed during the current study are available from the corresponding author on reasonable request.

## Abbreviations

MRI: Magnetic resonance imaging
ICC: Intra class correlation coefficient
CT: Computed tomography
FDG: Fluorodeoxyglucose
PET: Positron emission tomography
IMWG: International Myeloma Working Group
WB: Whole body
DW: Diffusion weighted
ADC: Apparent diffusion coefficient
MY-RADS: Myeloma Response Assessment and Diagnosis System
PI: Proteasome inhibitor
IMiD: Immunomodulatory drug
LCO: Light chain only
NS: Non secretory
